# Physiologically Based Pharmacokinetic Modeling of Digoxin in Adult and Pediatric Patients with Heart Failure

**DOI:** 10.3390/pharmaceutics18010112

**Published:** 2026-01-15

**Authors:** Yicui Zhang, Yao Liu, Hua He, Kun Hao

**Affiliations:** 1 State Key Laboratory of Natural Medicine, Jiangsu Province Key Laboratory of Drug Metabolism and Pharmacokinetics, China Pharmaceutical University, Nanjing 210009, China; 2 Department of Pharmacy, Children’s Hospital of Nanjing Medical University, Nanjing 210009, China; 3 Center of Drug Metabolism and Pharmacokinetics, China Pharmaceutical University, Nanjing 210009, China

**Keywords:** PBPK, digoxin, pediatric heart failure

## Abstract

**Background/Objectives**: Digoxin is a cardiotonic agent with a narrow therapeutic window and a high risk of toxicity. The current clinical use is based on an empirically FDA-recommended regimen which has wide dosing ranges, introducing the risk of inappropriate dosing and related adverse events. This study aims to develop a physiologically based pharmacokinetic (PBPK) model to characterize digoxin pharmacokinetics in adult and pediatric patients with heart failure, and then to evaluate the FDA-recommended regimen. **Methods**: The PBPK model was initially developed in healthy adults using PK-Sim^®^. Then, it was translated to adults with heart failure by incorporating disease factors. Next, it was further translated to pediatrics by scaling age-related parameters. Finally, through two-step translations, the model was used to evaluate current dosing regimens to inform safety and effectiveness based on observing predicted trough concentrations at a steady state. **Results**: This PBPK model has strong predicting ability, where observed concentrations and key PK metrics (C_max_, AUC_0-t_) were within 0.5–2.0-fold of predictions in healthy adults, adults with heart failure, neonates, and infants. The model prediction work on the evaluation of recommended dosing regimens from the FDA shows that the current regimen may not achieve the lowest boundary of the therapeutic window (0.5–2 ng/mL) in neonates (0–30 days), whereas infants (1–2 months) and children (<18 years) are generally good within it. **Conclusions**: This PBPK model explained major physiological and pathological contributors to differences in digoxin pharmacokinetics across populations and showed good performance in pediatric extrapolation. It also pointed out the shortage of empirical dosing regimens for such a drug with a narrow therapeutic window. The model may assist in optimizing the pediatric dosing strategies of digoxin, and suggests that current neonatal dosing regimens need refinement.

## 1. Introduction

Digoxin is a cardiotonic drug commonly used in heart failure, including in pediatric patients. However, the narrow therapeutic window of digoxin (0.5–2 ng/mL) places patients at high risk of toxicity, potentially resulting in serum potassium disturbances and consequent arrhythmias [1,2,3]. Therefore, therapeutic drug monitoring (TDM) is used in clinical settings [4] to ensure the safe and effective dosing of digoxin with a narrow therapeutic window. In addition, inter-population physiological variability may further impact the safety profile of digoxin, such as renal function, age, complications, etc. Premature infants are especially sensitive to the pharmacological effects and toxicity, necessitating not only dose reduction but also careful individualization based on their developmental maturity.

Currently, pediatric dosing of digoxin is largely based on recommendations for drug labeling, which often provide broad dose ranges without addressing significant individual variability among children [5]. Pharmacokinetic (PK) studies in children are limited by ethical and practical constraints [6], resulting in scarce clinical data and making it difficult to fully characterize pediatric PK. In addition, among pediatric patients, critical individual-specific factors, such as renal function [7], severity of heart failure, hemodynamic changes, Na^+^/K^+^-ATPase expression levels, postnatal age, body weight, and height, vary substantially and require individualized dosing strategies.

Physiologically based pharmacokinetic (PBPK) modeling provides a mechanistic approach to overcome these challenges [8,9]. By integrating physiological and biochemical parameters, PBPK models quantitatively simulate drug absorption, distribution, metabolism, and excretion [6]. Patient-specific characteristics, including age, body weight, organ function (e.g., hepatic and renal clearance), and genetic variability in drug-metabolizing enzymes and transporters, can be incorporated to predict drug exposure across populations and capture inter-individual variability. This enables individualized simulation of digoxin pharmacokinetics and supports stepwise extrapolation to populations with limited clinical data, such as pediatric patients [10].

This study aims to develop a stepwise PBPK model bridging healthy adults, adults with heart failure, and pediatric patients with heart failure, to elucidate population-specific differences in digoxin. In addition, the model is used to evaluate the suitability of current clinical dosing regimens across different pediatric age groups.

## 2. Materials and Methods

### 2.1. Software

Plasma concentration–time profiles from the published literature were digitized using Engauge Digitizer (version 12.1, Engauge Software, Chicago, IL, USA). Pharmacokinetic modeling and simulation were conducted using the open-source platform PK-Sim^®^ (version 11.0, Open Systems Pharmacology, Leverkusen, Germany). Data visualization and plotting were performed using R (version 4.5.0, The R Foundation for Statistical Computing, Vienna, Austria) in RStudio (version 2025.09.2, RStudio, Inc., Boston, MA, USA).

### 2.2. Model Development

#### 2.2.1. Model Assumptions

**Figure 1:**
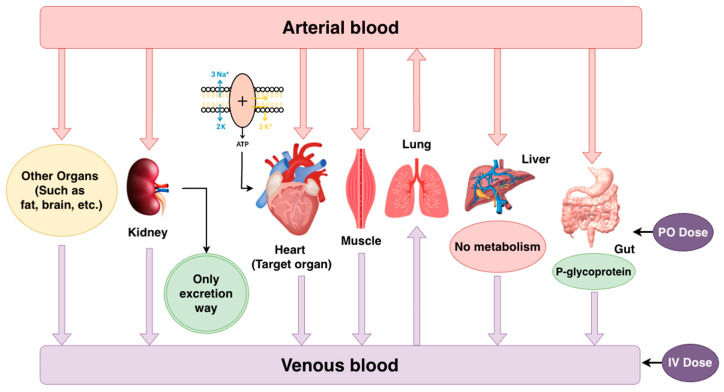
Schematic illustration of the digoxin PBPK model based on the assumptions. (Red arrows indicate arterial blood flow from the central compartment to individual organs, whereas purple arrows represent venous return to the central circulation).

To construct the PBPK model, several assumptions regarding the pharmacokinetics of digoxin were made based on established physiological and biochemical evidence. Figure 1 illustrates the digoxin PBPK model structure based on these assumptions.

- For absorption, digoxin was modeled as a substrate of P-glycoprotein (P-gp) [11,12], consistent with its known efflux by intestinal epithelial cells [13]. Its absorption was assumed and described using Michaelis–Menten kinetics [14].
- For distribution, it exhibits approximately 25% plasma protein binding [15], primarily to albumin [16], and has a strong tissue binding affinity, particularly to Na^+^/K^+^-ATPase in cardiac muscle [17], which is a key pharmacological target located on the membranes of excitable tissues. The binding process was assumed to be linear, non-saturated by the dissociation constant rate (Koff) and dissociation constant (Kd).
- For metabolism, digoxin was assumed not to undergo enzymatic biotransformation, in line with data showing that only approximately 13% of the drug is metabolized in vivo. It was assumed that there is no enzyme participating in digoxin’s metabolism.
- For excretion, renal excretion was assumed as the sole elimination pathway, due to the fact that 50–70% of digoxin is excreted unchanged in urine [18], with negligible biliary elimination. The “Glomerular Filtration Rate (GFR) fraction” chosen for digoxin was fixed to 1, indicating predominantly renal elimination via glomerular filtration [19].

#### 2.2.2. Stepwise Extrapolation Process

The workflow was conducted from healthy adults to children with disease through to adult patients, rather than healthy children, since clinical data used for validation from healthy children are ethically hard to obtain, whereas diseased adults’ data are more available.

**Figure 2:**
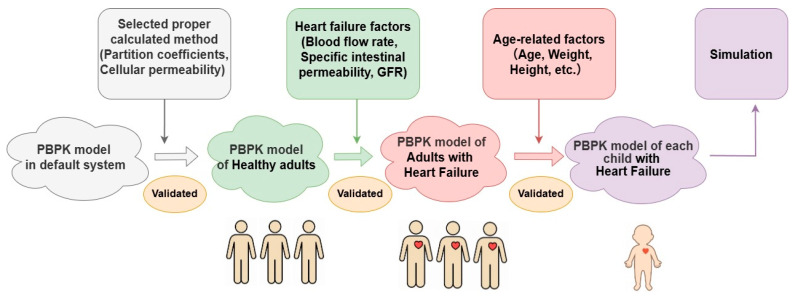
Stepwise extrapolation framework of the PBPK modeling process from healthy adults to pediatric heart failure populations. (Note that adult PBPK models were developed using population-averaged physiological and biochemical parameters, whereas pediatric PBPK models were constructed on an individual basis to account for large inter-individual variability in age, body weight, and growth.).

A stepwise extrapolation process was applied (Figure 2). In the first translation, physiological changes associated with heart failure—such as alterations in organ blood flow and intestinal permeability—were incorporated to generate an adult heart failure PBPK model. After validation, the second translation was performed by further extrapolating adult heart failure populations to pediatric populations by adjusting for age-specific physiological differences, including body size, renal function, developmental parameters, etc. The established model can predict drug exposure based on individual pediatric physiological characteristics, thereby facilitating dose adjustment. Parameter fitting was performed either manually or using the built-in parameter identification module in PK-Sim^®^, which applies a Monte Carlo algorithm to optimize parameters and approximate the observed plasma concentration–time profiles.

### 2.3. Model Validation

Visual inspection: The model should consistently reproduce the general trend of the data [20], which means for each population, the predicted simulation line should be visually aligned well with the observed data.

Then, model accuracy was evaluated by assessing whether the observed data fell within the 0.5- to 2-fold range. In addition, if key pharmacokinetic parameters (AUC_0–t_ and C_max_) also fell within this range, the model was considered to have good predictive performance.

(1) $$Ratio=\frac{{PK\;parameter}_{observed}}{{PK\;parameter}_{predicted}}$$

### 2.4. Sensitivity Analysis

A local sensitivity analysis was performed to evaluate the influence of individual input parameters on selected pharmacokinetic (PK) outputs. The sensitivity of a PK parameter (${PK}_{j}$) with respect to an input parameter ($p_{i}$) was calculated using the following equation:

(2) $${Sensitivity\;}_{i,j}=\frac{∆{PK}_{j}}{∆p_{i}}\cdot \frac{p_{i}}{{PK}_{j}}\;$$

∆*p_i_* represents a small perturbation of the input parameter, and $∆{PK}_{j}$ is the resulting change in the PK output following a simulation with the perturbed input, while all other parameters were held constant.

This dimensionless sensitivity coefficient quantifies the relative change in PK output in response to a relative change in an input parameter. For example, a sensitivity of −1.0 indicates that a 10% increase in the input parameter leads to a 10% decrease in the PK output. Conversely, a sensitivity of +0.5 means that a 10% increase in the input results in a 5% increase in the PK output.

### 2.5. Model Prediction

According to the FDA-approved prescribing information for Lanoxin (digoxin injection) digoxin [3], administration in children typically follows a two-phase regimen, consisting of an initial loading dose (also referred to as digitalization) followed by a maintenance dose. The purpose of the loading dose is to rapidly raise serum digoxin concentrations to a therapeutic level to ensure a prompt pharmacologic effect.

The recommended protocol involves administering a total loading dose equal to the full digitalization amount, delivered in two divided doses over a 6–8 h interval. Maintenance dosing begins 12 h after the loading dose and is administered twice daily, with each dose representing 25% of the total loading dose. Intravenous (IV) administration is used at 75% of the corresponding oral dose.

Pediatric dosing was categorized into three age groups: neonates (0–30 days), infants (1 month–2 years), and children older than 2 years. Since dosing recommendations are generally consistent for children above 2 years of age, and their physiological characteristics are relatively stable with less variability compared with infants and newborns, they were grouped into a single category.

In this study, each pediatric subgroup received two dosing levels: a low dose (at the lower end of the recommended range) and a high dose (at the upper end of the range). For example, in the term neonates, the low-dose group received 0.02 mg/kg orally, while the high-dose group received 0.03 mg/kg. Each group included both oral and intravenous administration simulations. Dosing regimens are shown in Table 1.

The criteria of evaluation for children’s TDM in clinical practice for digoxin is to observe whether the trough concentration at steady state (Cmin,ss) is in the therapeutic window (0.5–2 ng/mL). If Cmin,ss is within it, we can conclude that this kind of dosing regimen is effective and acceptable for children. If it is outside digoxin’s therapeutic window, the existing regimens should be adjusted for a certain age group.

## 3. Results

### 3.1. Model Extrapolation Process

#### 3.1.1. Model Development of Healthy Adults

IV fitting for digoxin mainly focused on the distribution part, since there is no metabolism for digoxin based on our assumptions, and the only excretion method is renal clearance using the ‘GFR fraction’, which is 1, meaning that all digoxin is delivered to the kidney through filtration to be cleared [21].

LogP, fu, solubility, and pKa were initially obtained from ‘Drugbank’ (https://go.drugbank.com/drugs/DB00390, accessed on 1 October 2022). Since those values are from different resources, we optimized these values within a reasonable range for healthy adults. fu was given a range from 0.7 to 0.8, then we used 0.71, which is reasonable. LogP is fitted to 2.36, within a range that is from 1.04, calculated by ALOGPS, to 2.37, calculated by Chemaxon. “Specific organ permeability” represents cellular permeability, which was initially 1.01 × 10^−4^ cm/min, calculated by fitted LogP using Rodgers & Rowland’s method, then slightly optimized to 3 × 10^−4^ cm/min.

Digoxin is a BCS IV-classified drug with low solubility and low permeability due to P-gp in the intestine. The solubility is 0.0648 mg/mL from experiment, and 0.127 mg/mL calculated by ALOGPS. Even though solubility has a measured value, since the experimental value of 0.0648 mg/mL was measured under a certain condition (25 °C), and its solubility is very low (<0.2 mg/mL), giving it huge variability, it may be not the best option to use directly in a model for a complex physiological environment. Based on that, the values were fitted to 0.1 mg/mL, using the range from 0.0648 to 0.0127 mg/mL. PO fitting focused on P-gp and intestinal permeability. P-gp Km was obtained from Troutman‘s study [22], and Vmax was fitted to 20 µmol/L/min. Intestinal permeability was also optimized based on PO observed data, finally reaching 2.3 × 10^−5^ cm/min. All the fitted values are shown in Table 2.

Then, the physiochemical parameters (LogP, fu, solubility, pKa, and MW) are kept the same among the three populations, and the cellular permeability as well. The final parameter set is summarized in Table 2.

#### 3.1.2. From Healthy Adults to Adults with Heart Failure

The model of adults with heart failure was constructed based on a digoxin healthy adult model incorporating physiological changes in heart failure. There are several considerations in the first translation from healthy adults to adults with heart failure.

In patients with heart failure, systemic blood flow rate is reduced in proportion to the severity of heart failure. In this model, systemic blood flow was reduced by half, as Sullivan and coworkers reported around 50% reduction in systemic blood flow in patients with moderate chronic heart failure [24]. The blood flow rate of healthy adults was set using the default value in PK-Sim, then manually halved to adapt to adults with heart failure. For example, the blood flow rate of healthy adults is 1.46 L/min in the kidney, which was then adjusted to 0.73 L/min in adult patients.

“Specific intestinal permeability” was increased in those with heart failure, since evidence shows that intestinal mucosal ischemia in heart failure can increase intestinal permeability and bacterial translocation [25]. Then, the value is converted to 2.7 × 10^−5^ cm/min from 2.3 × 10^−5^ cm/min.

GFR tends to decline accordingly during the progression of heart failure. Gilbert’s study [25] suggests that for digoxin, GFR may decrease by approximately 50–60% in patients with moderate to severe heart failure. Accordingly, GFR was adjusted from 116.45 to 43.85 mL/min in the patients’ group.

During the onset of heart failure, both rodents and humans exhibit reduced expression [26,27] of sodium pump affinity (Na+/K+-ATPase) and receptor isoforms in cardiac tissues. There are several α-subunit isoforms (α1, α2, α3, and α4) [28], which are tissue-specific and species-dependent. Among them, isoforms α2 and α3 are associated with high affinity for digoxin [29]. A reduction in their expression would significantly reduce digoxin binding and uptake into cardiac cells. In patients with heart failure, this could result in up to a 40% decrease in intracellular digoxin concentrations [30]. Therefore, in PBPK modeling, the dissociation constant (Kd) of digoxin should be fitted from 0.01 to 0.5 accordingly to reflect decreased affinity. The final parameter set is summarized in Table 2.

#### 3.1.3. From Adults with Heart Failure to Pediatric Patients

The model of pediatrics with heart failure was translated from adult patients. During the second translation, age-dependent physiological parameters were mainly considered in pediatrics.

Blood flow rate for each organ should be decreased due to the smaller organ size in children. This step was scaled automatically by the ‘scaling’ function of PK-Sim^®^ for each child. For example, the blood flow rate of the kidney is 0.07 L/min after scaling.

Renal function differs significantly from that of adults, due to the reduced blood flow rate and GFR [31]. These values were also scaled from heart failure adults to pediatric subjects using ‘scaling’ in PK-Sim^®^. Now, GFR is 4.2 L/min.

For Na^+^/K^+^-ATPase activity, young animals have higher myocardial Na^+^/K^+^-ATPase activity [32]. This is because the inhibition of Na^+^/K^+^-ATPase is an action of the mechanism of digoxin, which may underlie its increased tolerance to digitalis glycosides, and explain why neonates and infants may require higher doses of digoxin to achieve comparable pharmacodynamic and toxicodynamic effects. For tissue binding, evidence [32] also shows that, despite similar plasma concentrations, infants and young children exhibit substantially higher myocardial accumulation of digoxin compared with adults. Therefore, the Kd value was reduced by fitting from 0.5 to 0.005 to reflect the changes in digoxin in children compared to adults.

Since only IV observed data were obtained from neonates and infants, P-gp-related parameters kept the same and will not influence pediatric IV model. Intestinal permeability kept the same as well. The final parameter set is summarized in Table 2.

### 3.2. Model Validation

**Figure 3:**
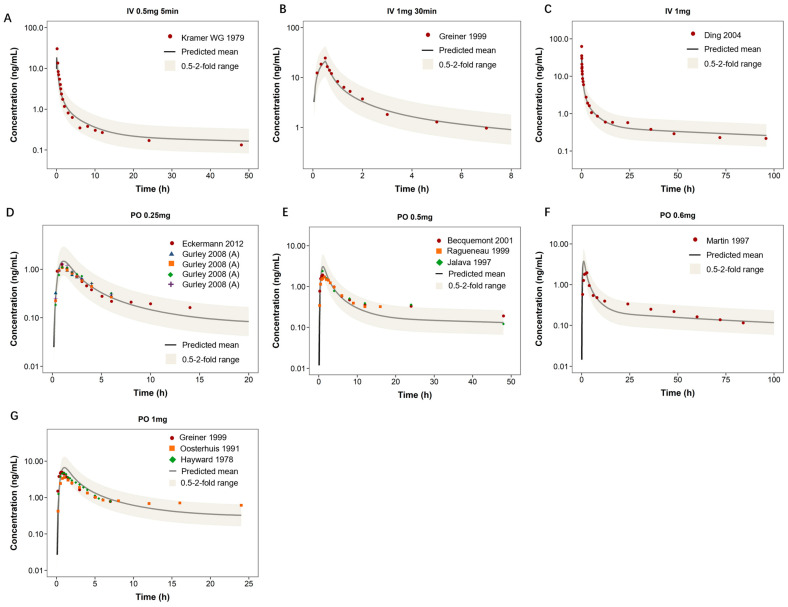
Predicted and observed plasma concentration–time profiles of digoxin in healthy adults. This figure presents a comparison between predicted and observed plasma concentration–time profiles of digoxin in healthy adult subjects under different dosing regimens. Panels (**A**–**C**) illustrate intravenous (IV) administration at various doses and infusion durations, while panels (**D**–**G**) depict oral (PO) dosing. The solid black line indicates the model-predicted mean concentration, and the shaded area shows the 0.5- to 2-fold prediction range. Red dots represent observed clinical data from literature.

As shown in Figure 3, the PBPK model predicted digoxin plasma concentration–time profiles across a range of intravenous (IV) and oral (PO) regimens in healthy adults. For IV dosing (Figure 3A–C), the model captured the rapid distribution and biexponential decline, with observed data falling within the 0.5- to 2-fold prediction range. For oral regimens (Figure 3D–G), the model adequately described absorption and distribution patterns, with good agreement between predicted and observed concentrations across doses ranging from 0.25 to 1 mg. The predicted AUC _0–t_ and C _max_ values were generally within two-fold of observed values (Table 3). Most predicted AUC _0–t_ values were within 0.9–1.0 times the observed values, indicating good model performance in capturing systemic exposure.

**Figure 4:**
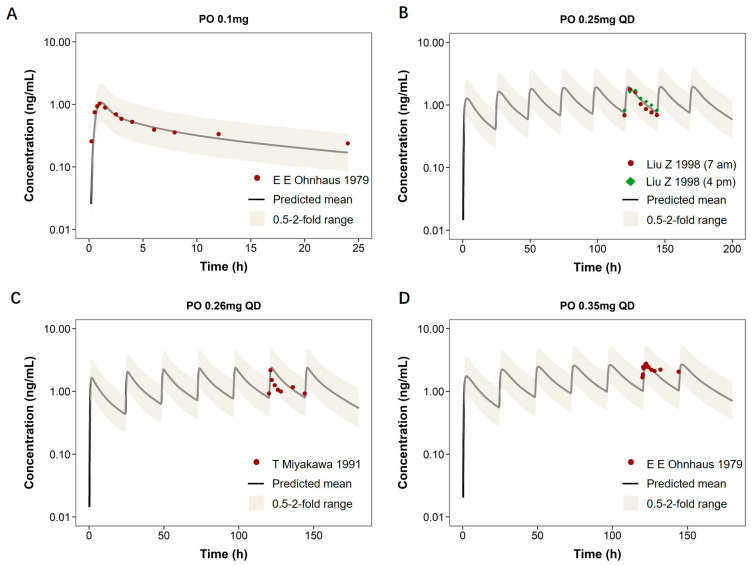
Predicted and observed plasma concentration–time profiles of digoxin in adults with heart failure. Plasma concentration–time profiles of digoxin in adults with heart failure are shown under different dosing regimens. Panel (**A**) illustrates a single oral dose (0.1 mg), while panels (**B**–**D**) depict multiple-dose regimens (0.25–0.35 mg, QD). The solid black line represents model-predicted mean concentration, and the shaded area indicates the 0.5- to 2-fold prediction range. Red and green markers correspond to observed clinical data from published studies.

The model was further extrapolated to adults with heart failure by incorporating pathophysiological changes such as altered renal function and tissue perfusion. As shown in Figure 4, the predicted concentration–time curves for both single and multiple oral doses (0.1–0.35 mg) closely matched the observed data. The predicted AUC _0–t_ values were generally consistent with observed data, with prediction-to-observation ratios ranging from 0.84 to 1.30. For C_max_, the predicted values also demonstrated reasonable accuracy, with most ratios ranging from 0.88 to 1.03, supporting the model’s applicability in diseased adults.

**Figure 5:**
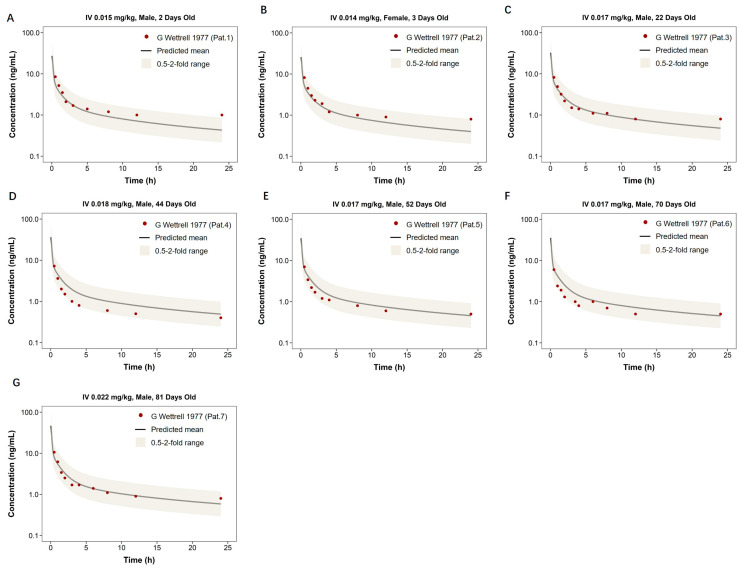
Predicted and observed plasma concentration–time profiles of digoxin in pediatric heart failure patients. This figure displays the plasma concentration–time profiles of digoxin in individual pediatric patients with heart failure following intravenous administration. All panels (**A**–**G**) represent different patients aged 2 to 81 days, with doses ranging from 0.014 to 0.022 mg/kg. The solid black line shows the model-predicted mean concentration, and the shaded area indicates the 0.5- to 2-fold prediction range. Red dots denote observed clinical data digitized from published literature [33].

Pediatric model extrapolation incorporated age-dependent physiological changes. Figure 5 shows model predictions for neonates and infants (2–81 days old) receiving IV digoxin at doses of 0.014–0.022 mg/kg. The model successfully captured the observed plasma concentration–time profiles across all subjects. For AUC_0–t_, the observed-to-predicted ratios ranged from 0.59 to 1.23, with most values falling between 0.6 and 0.9. For C_max_, the ratios ranged from 0.54 to 0.87 across individuals. The variability in predictions may be attributed to age-related physiological differences and model assumptions in this sensitive population. As summarized in Table 3, the predicted AUC_0–t_ and C_max_ values were within the two-fold range, indicating satisfactory predictive performance in this vulnerable population.

**Table 3:** Observed and predicted AUC _0–t_ and C_max_ of digoxin in different populations.

Population	Administration	AUC_0−t_ (ng·h/mL)			Cmax (ng/mL)			References
		Obs ^1^	Pred ^1^	Ratio	Obs	Pred	Ratio	
Healthy adults	IV, 0.5 mg, 5 min	21.86	22.43	0.97	30.28	18.09	1.67	[34]
	IV, 1 mg, 30 min	57.02	58.06	0.98	62.69	38.00	1.65	[35]
	IV, 1 mg	26.38	26.76	0.99	24.59	21.24	1.16	[36]
	PO, 0.25 mg	3.45	5.77	0.60	1.11	1.47	0.76	[37,38]
	PO, 0.5 mg	19.99	15.87	1.26	1.92	3.08	0.62	[39,40,41]
	PO, 0.6 mg	25.94	26.23	0.99	1.96	3.76	0.52	[42]
	PO, 1 mg	13.32	25.80	0.52	4.98	6.64	0.75	[35,43,44]
Adults with Heart Failure	PO, 0.1 mg	9.23	8.30	1.11	1.03	1.07	0.96	[45]
	PO, 0.25 mg	30.31	30.16	1.00	1.71	1.95	0.88	[46]
	PO, 0.26 mg	27.39	32.73	0.84	2.17	2.40	0.90	[47]
	PO, 0.35 mg	53.3	41.01	1.30	2.76	2.67	1.03	[45]
Pediatrics with Heart Failure	IV 0.015 mg/kg, Male, 2 Days Old	32.3	26.29	1.23	8.5	10.22	0.83	[33]
	IV 0.014 mg/kg, Female, 3 Days Old	28.42	24.41	1.16	8.2	9.45	0.87	
	IV 0.017 mg/kg, Male, 22 Days Old	28.05	29.16	0.96	8.2	11.42	0.72	
	IV 0.018 mg/kg, Male, 44 Days Old	17.52	29.92	0.59	7.2	11.92	0.60	
	IV 0.017 mg/kg, Male, 52 Days Old	20.77	27.95	0.74	7.0	11.22	0.62	
	IV 0.017 mg/kg, Male, 70 Days Old	18.05	27.5	0.66	6.0	11.2	0.54	
	IV 0.022 mg/kg, Male, 81 Days Old	31.68	35.49	0.89	10.6	14.55	0.73	

^1^ Obs: observed; Pred: predicted.

### 3.3. Sensitivity Analysis

**Figure 6:**
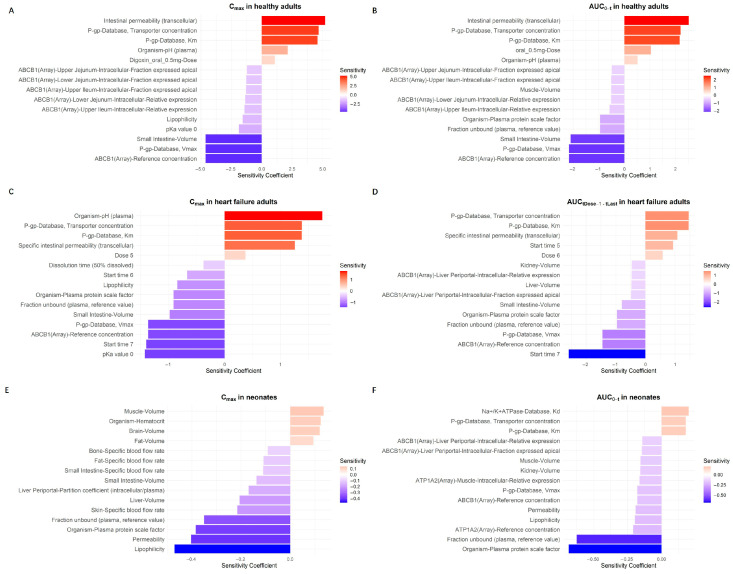
Sensitivity analysis of model parameters for digoxin C_max_ and AUC in different populations. This figure presents the results of local sensitivity analyses evaluating the influence of physiological parameters on model outputs in three populations: healthy adults (Panels (**A**,**B**)), adults with heart failure (Panels (**C**,**D**)), and neonates (Panels (**E**,**F**)). Panels (**A**,**C**,**E**) correspond to the maximum plasma concentration (C_max_), while Panels (**B**,**D**,**F**) depict the area under the concentration–time curve (AUC). The bars represent sensitivity coefficients calculated based on parameter perturbation. Positive and negative values indicate direct or inverse relationships with the corresponding PK outputs.

A local sensitivity analysis was conducted to identify model parameters that most strongly influenced digoxin exposure (AUC) and peak concentration (C_max_) across populations (Figure 6). In healthy adults, intestinal permeability and P-gp-related parameters (transporter concentration and Km) had the greatest positive influence on both C_max_ and AUC (Figure 6A,B). Parameters such as small intestinal volume, fraction unbound, and relative expression of ABCB1 transporters had negative sensitivity coefficients, indicating an inverse effect on drug exposure.

In heart failure patients (Figure 6C,D), P-gp transporter parameters remained key contributors to variability in both C_max_ and AUC. P-gp-related parameters, including transporter concentration, Km, and Vmax, were identified as highly influential in the sensitivity analysis of both C_max_ and AUC. This includes the ABCB1 (reference concentration), which corresponds to the gene encoding P-glycoprotein. These findings highlight the significant impact of P-gp expression and function on the pharmacokinetics of digoxin in heart failure patients. Additionally, specific intestinal permeability and dissolution time were also influential. Conversely, parameters such as organ-specific volumes and fraction unbound exerted negative effects on model outputs.

In neonates (Figure 6E,F), the sensitivity profile shifted. In pediatric patients, digoxin is primarily administered via intravenous injection rather than oral routes. As a result, parameters related to intestinal absorption, such as P-gp transporter activity, did not appear among the most sensitive factors in the local sensitivity analysis. This reflects the limited relevance of efflux transporters like P-gp under intravenous dosing conditions. Organ-specific physiological parameters, such as liver and muscle volume, brain blood flow, and fraction unbound in plasma, played a more prominent role in determining digoxin exposure.

These results highlight population-specific determinants of digoxin pharmacokinetics and support the model’s utility in guiding dose adjustments based on individual physiological and transporter-related variability.

### 3.4. Model Prediction

**Figure 7:**
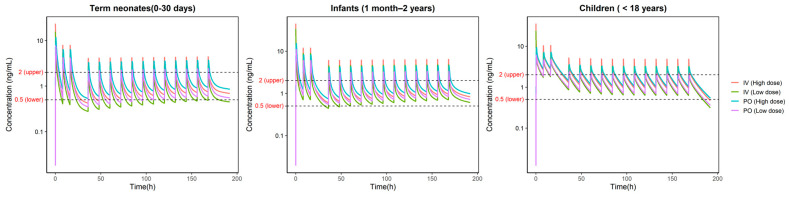
Predicted pharmacokinetic profiles under FDA dosing regimens of digoxin in pediatric populations of different age groups: term neonates (0–30 days), infants (1 month–2 years), and children (<18) years. The therapeutic window is indicated by dashed lines (0.5–2 ng/mL).

Based on the criteria of evaluation for children’s TDM, it is observed that the trough concentration at steady state (Cmin,ss) should be in therapeutic window (0.5–2 ng/mL). Figure 7 presents the predicted pharmacokinetic concentration–time profiles of digoxin under standard dosing regimens across three pediatric age groups. Even though high peak concentrations (C max) were observed in these groups, it is still considered irrelevant, because digoxin is an effect-site delay drug, and its toxicity is not directly correlated with the plasma peak concentration, which is also sharply decreased after reaching the peak.

In the term neonates’ group (0–30 days), the steady-state trough concentrations in both IV and PO low-dose regimens were below the lower limit of the therapeutic range, suggesting that this dosing strategy may not provide adequate therapeutic exposure. High-dose groups were generally satisfactory.

In the infant group (1 month–2 years), at the beginning stage of administration, the low-dose IV group was reaching the lower boundary of therapeutic window, suggesting that it may more slowly achieve a therapeutic effect. For rest of the three dosing groups, the performance was generally good.

In the childrens’ group (2–18 years), all dosing regimens exhibited relatively stable concentration–time profiles with overall exposure and less fluctuation. Most regimens remained within the therapeutic window, particularly the oral low-dose regimen, which maintained plasma concentrations within the therapeutic range throughout the simulation, suggesting favorable safety. However, the gap between PO and IV seems smaller than younger groups, suggesting the current method of converting the IV dose directly from 75% of the corresponding PO dose may need reconsidering for younger children’s groups.

## 4. Discussion

Pediatric heart failure has numerous challenges in treatment, with insufficient pharmacokinetic studies and significant physiological changes during development. Children are not simply “small adults” [48], and they need more accurate dosing and physiological analysis.

This PBPK model provides a mechanistical method to dig into the principle of pediatric PK. Through a stepwise extrapolation from healthy adults to adults with heart failure, and then to pediatric patients, the model characterized PK differences in numerous angles. In the first translation, disease-related changes were represented as systematic blood flow rate, intestinal permeability, Na^+^/K^+^-ATPase affinity, and GFR. In the second translation, age-related parameters were considered, including scaled physiology (body weight, organ size, blood flow rate, etc.), deficient renal function, and increasing Na^+^/K^+^-ATPase affinity. Those factors significantly contribute to PK differences across populations. Therefore, the final version of the PBPK model can provide accurate simulations and predictions when applied in pediatrics.

The model prediction results also reveals that in term neonates, the intravenous low-dose current regimen led to trough concentrations below the lower limit of the therapeutic window, suggesting that the currently used standard dosing may fail to achieve optimal therapeutic exposure. Importantly, our mechanistic framework allows for the elucidation of the underlying cause, the increasing Na^+^/K^+^-ATPase affinity in neonates, which mainly contributes to their closer binding with tissue with lower plasma concentration. Consequently, neonates and infants may require higher doses to achieve equivalent pharmacodynamic and toxicodynamic effects. This finding reflects the maturation of drug disposition processes with age and represents the limitations of uniform dosing strategies in pediatric populations. Thus, this study supports dose adjustments based on the developmental stage to achieve proper efficacy.

The existing PK models of digoxin barely consider pediatric situations based on developmental physiology. Compared to traditional population pharmacokinetic (PopPK) models [49,50] or empirically scaled dosing strategies [3] that primarily rely on data availability to characterize observed PK behavior, this PBPK model enables reliable extrapolation and prediction across age groups with data scarcity, and also provides physiological explanations of PK differences between adults and children. Beyond dose adjustment [8,9], the PBPK model also has advantages in predicting drug–drug interactions [51], supporting formulation evaluation [52], and informing regulatory decisions [53]. With further integration of real-world data, population variability modeling, and prospective clinical validation, this model may expand its utility in both pediatric pharmacotherapy and regulatory science [53].

Several limitations should also be pointed out in this model with limited pediatric data. Due to ethical constraints [6] and the inherent challenges of conducting clinical studies in children, pediatric pharmacokinetic data of digoxin remain limited. In this paper, there are only seven neonates and infants obtained for us to characterize their digoxin PK behavior and perform validation. Data from other age groups are still unavailable to obtain for validating our simulations. In the future, more observed data from different age groups could enrich and improve this work. Next, digoxin is a substrate of P-gp [49], yet the expression and activity levels of P-gp during various stages of pediatric development are not well characterized. This is because there is only limited IV observed data from neonates and infants; we could not optimize the influence of P-gp changes without PO observed PK performance. In addition, numerous drug–drug interactions (DDIs) mediated by P-gp have been studied [15,22,49,51,54], whereas the current model focuses solely on digoxin monotherapy rather than co-medications. Future work could focus on pediatric DDI models if data for validation is available. Moreover, heart failure has different disease progressions [55], but the current model did not classify patients by disease severity, since the paper containing PK data did not clearly illustrate all the levels of heart failure. This may limit its ability to reflect pathophysiological variability among different subgroups. Lastly, the model focuses solely on the pharmacokinetics of digoxin and does not incorporate pharmacodynamic (PD) components related to therapeutic effects or toxicity, such as changes in heart rate or the risk of arrhythmias.

Future research could focus on clinical applicability. Integration of PD components, particularly linking predicted cardiac tissue concentrations with clinical outcomes, will enable a more comprehensive PK–PD framework. Moreover, expanding the model to include different pediatric subgroups—such as varying stages of heart failure, comorbidities like renal impairment, or alternative dosing routes—will further enhance its utility. Apart from that, the integration of PBPK modeling with machine learning techniques also offers promising opportunities for automated parameter optimization and improved model scalability.

## 5. Conclusions

This study developed a PBPK model to characterize digoxin pharmacokinetics in pediatric heart failure patients by incorporating age-related development and disease-specific physiology through two stepwise extrapolations. Using this model, current pediatric dosing recommendations were evaluated, and their potential inadequacy in neonates was identified. By integrating developmental physiology, this model provides a strong reference to support dose adjustment and optimization strategies in clinical practice.

## Figures and Tables

**Table 1:** Recommended PO and IV loading doses of digoxin in pediatric populations for simulation.

Age Groups	PO Loading Dose (mg/kg)	IV Loading Dose (mg/kg)
Term neonates (0–30 days)	0.02–0.03	0.0150–0.0225
Infants (1 month–2 years)	0.03–0.04	0.0225–0.0300
Children (2–18 years)	0.02–0.03	0.0150–0.0225

**Table 2:** Parameters for the Digoxin PBPK Model.

Parameters	Initial Value	Fitted Value		
	Healthy Adults	Healthy Adults	Adults with HF	Children with HF (e.g., 3-Day-Old)
**Physiological Parameters**				
Blood flow rate (L/min)-Bone	2.42 ^b^	2.42 ^b^	1.21 ^c^	0.02 ^c^
Blood flow rate (L/min)-Brain	0.68 ^b^	0.68 ^b^	0.34 ^c^	0.09 ^c^
Blood flow rate (L/min)-Fat	0.28 ^b^	0.28 ^b^	0.14 ^c^	0.02 ^c^
Blood flow rate (L/min)-Gonads	2.84 × 10^−3 b^	2.84 × 10^−3 b^	1.42 × 10^−3 c^	6.07 × 10^−5 c^
Blood flow rate (L/min)-Heart	0.22 ^b^	0.22 ^b^	0.11 ^c^	0.01 ^c^
Blood flow rate (L/min)-Kidney	1.46 ^b^	1.46 ^b^	0.73 ^c^	0.06 ^c^
Blood flow rate (L/min)-Large intestine	0.26 ^b^	0.26 ^b^	0.13 ^c^	0.01 ^c^
Blood flow rate (L/min)-Small intestine	0.65 ^b^	0.65 ^b^	0.33 ^c^	0.03 ^c^
Blood flow rate (L/min)-Liver	0.46 ^b^	0.46 ^b^	0.23 ^c^	0.02 ^c^
Blood flow rate (L/min)-Muscle	0.98 ^b^	0.98 ^b^	0.49 ^c^	0.02 ^c^
Blood flow rate (L/min)-Pancreas	0.06 ^b^	0.06 ^b^	0.03 ^c^	3.05 × 10^−3 c^
Blood flow rate (L/min)-Skin	0.28 ^b^	0.28 ^b^	0.14 ^c^	0.02 ^c^
Blood flow rate (L/min)-Spleen	0.14 ^b^	0.14 ^b^	0.07 ^c^	7.76 × 10^−3 c^
Blood flow rate (L/min)-Stomach	0.06 ^b^	0.06 ^b^	0.03 ^c^	3.04 × 10^−3 c^
Blood flow rate (L/min)-GIT-mucosa ^1^ (e.g., Duodenum)	0.02 ^b^	0.02 ^b^	0.012 ^c^	2.12 × 10^−3 c^
GFR (mL/min)	116.45 ^b^	116.45 ^b^	43.85 ^c^	4.2 ^c^
**Physicochemical Parameters**				
Log P	2.37 ^a^, 1.04 ^a^	2.36 ^c^	2.36	2.36
fu	0.7–0.8 ^a^	0.71 ^c^	0.71	0.71
MW (g/mol)	780 ^a^	780	780	780
pKa (acid)	7.15 ^a^	7.15	7.15	7.15
Solubility (mg/mL)	0.0648 ^a^, 0.127 ^a^	0.1 ^c^	0.1	0.1
**Drug-specific Parameters**				
Specific intestinal permeability (cm/min)	2.76 × 10^−6 b^	2.3 × 10^−5 c^	2.7 × 10^−5^	2.7 × 10^−5^
Specific organ permeability (cm/min)	1.01 × 10^−4 b^	3 × 10^−4 c^	3 × 10^−4^	3 × 10^−4^
Na^+^/K^+^-ATPase-Koff (1/min)	1 × 10^−3^ [23]	1 × 10^−3^	1 × 10^−3^	1 × 10^−3^
Na^+^/K^+^-ATPase-Kd (µmol/L)	0.01 ^c^	0.01 ^c^	0.5 ^c^	0.005 ^c^
P-gp Vmax (µmol/L/min)	20 ^c^	20 ^c^	20	20
P-gp Km (µM)	177 [22]	177	177	177
GFR fraction	1 [21]	1	1	1
Calculation methods	Partition coefficients		Rodgers and Rowland	
	Cellular permeability		PK-Sim Standard	

^a^ from DrugBank. ^b^ Default value or Calculated by default algorithm in PK-Sim^®^. ^c^ Fitted. ^1^ Blood flow rate for GIT-mucosa (includes duodenum, upper jejunum, lower jejunum, upper ileum, lower ileum, cecum, colon and rectum) all changed as same percentage as duodenum.

## Data Availability

All data used in this study are available from the published literature cited in this article.
